# Prognostic significance and survival benefits of postoperative adjuvant chemotherapy in patients with stage IA lung adenocarcinoma with non-predominant micropapillary components

**DOI:** 10.1186/s12957-024-03303-x

**Published:** 2024-01-25

**Authors:** Rongyang Li, Jianhao Qiu, Zhenyi Li, Haiming Li, Zhanpeng Tang, Wenhao Yu, Hui Tian, Zhenguo Sun

**Affiliations:** Department of Thoracic Surgery, Qilu Hospital of Shandong University, Jinan, 250012 Shandong China

**Keywords:** Lung adenocarcinoma, Micropapillary component, Adjuvant chemotherapy, Prognosis, Survival

## Abstract

**Background:**

The prognostic significance of adjuvant chemotherapy (ACT) for patients with stage IA micropapillary non-predominant (MPNP) lung adenocarcinoma (LUAD) remains unknown. This study aimed to investigate the effects of postoperative ACT in patients with stage IA MPNP-LUAD.

**Methods:**

A total of 149 patients with pathological stage IA MPNP-LUAD who underwent surgery at our center were retrospectively analyzed. Propensity score matching (PSM) analysis was conducted to reduce potential selection bias. Kaplan–Meier analyses were used to assess the impact of ACT on recurrence-free survival (RFS), overall survival (OS), and disease-specific survival (DSS). Subgroup analyses were performed for the survival outcomes based on the percentage of micropapillary components. Cox proportional hazards regression analyses were applied to identify risk factors associated with survival.

**Results:**

The receipt or non-receipt of postoperative ACT had no significant effect on RFS, OS, and DSS among all enrolled patients with stage IA MPNP-LUAD (*P* > 0.05). For patients with a micropapillary component > 5%, the 5-year rates of RFS, OS, and DSS were significantly higher in the ACT group compared to the observation group, both before and after PSM (*P* < 0.05). However, the differences between the two groups were not significant for patients with a micropapillary component ≤ 5% (*P* > 0.05). The resection range (HR = 0.071; 95% CI: 0.020–0.251; *P* < 0.001), tumor size (HR = 2.929; 95% CI: 1.171–7.330; *P* = 0.022), and ACT (HR = 0.122; 95% CI: 0.037–0.403; *P* = 0.001) were identified as independent prognostic factors for RFS through Cox regression analysis.

**Conclusion:**

Patients with stage IA MPNP-LUAD who have a micropapillary component greater than 5% might benefit from postoperative ACT, while those with a micropapillary component ≤ 5% did not appear to derive the same benefit from postoperative ACT.

**Supplementary Information:**

The online version contains supplementary material available at 10.1186/s12957-024-03303-x.

## Background

Non-small cell lung cancer (NSCLC) remains a significant global health concern characterized by high rates of both incidence and mortality [1]. Among the various histological types, lung adenocarcinoma (LUAD) continues to be the most prevalent type [2]. At present, surgical resection remains the optimal therapeutic approach for the management of early-stage NSCLC and is associated with satisfactory survival outcomes [3, 4]. Based on the classification system established by the International Association for the Study of Lung Cancer (IASLC), American Thoracic Society (ATS), and European Respiratory Society (ERS), LUAD can be classified into several subtypes, including acinar, lepidic, solid, papillary, micropapillary, and invasive mucinous adenocarcinoma [5]. The micropapillary pattern is distinguished by the growth of tumor cells in papillary tufts that do not possess fibrovascular cores [6]. It has been reported that LUAD with micropapillary components is associated with an increased risk of lymph node metastasis and a more unfavorable prognosis, even in the early stages of the disease [7–10].

Adjuvant chemotherapy (ACT) plays a crucial role in the multidisciplinary management of NSCLC, significantly contributing to improving prognosis and prolonging survival in patients with advanced stages [11]. Nevertheless, previous studies have consistently demonstrated no survival benefit of postoperative ACT in patients with stage IA NSCLC [12, 13]. Consequently, the administration of ACT for patients at stage IA was not regularly recommended according to the National Comprehensive Cancer Network (NCCN) Clinical Practice Guidelines for NSCLC [14]. In recent years, several studies have indicated that postoperative ACT could provide survival benefits for patients with stage IA and IB LUAD who have a micropapillary predominant (MPP) pattern [15, 16]. In fact, the prevalence of the micropapillary non-predominant (MPNP) pattern (also known as micropapillary minor pattern) is more common than that of MPP adenocarcinoma, especially in stage IA LUAD [17]. A recently published meta-analysis indicated that the presence of a micropapillary component in stage IA LUAD was correlated with an increased risk of recurrence [10]. However, the potential survival benefits of ACT for patients with stage IA MPNP-LUAD have not been studied to date.

In this study, we aimed to investigate the clinical significance of postoperative ACT in patients with stage IA LUAD with non-predominant micropapillary components. Additionally, we sought to identify the specific subgroup of stage IA MPNP-LUAD patients who would benefit more from postoperative ACT.

## Patients and methods

### Study population

We conducted a retrospective search of the prospectively maintained database at Qilu Hospital of Shandong University to collect data on patients who underwent surgery for stage IA LUAD from January 2012 to December 2019. The inclusion criteria were as follows: 1) patients with postoperative pathologically confirmed MPNP-LUAD; 2) pathological tumor-node-metastasis (pTNM) stage was classified as stage IA according to the 8th TNM classification system; 3) patients aged ≥ 18 years; 4) patients with active follow-up information and detailed medical records. Patients who received adjuvant targeted therapy or radiotherapy, patients diagnosed with multiple primary lung cancer, and patients with incomplete clinicopathological information were excluded from this study. This retrospective study was approved by the Institutional Review Board (IRB) of Qilu Hospital of Shandong University, and a waiver of informed consent was obtained due to the retrospective nature of the study.

### Data collection and variable definitions

The following clinicopathological data of enrolled patients were collected from the database of Qilu Hospital of Shandong University: age, sex, smoking history, surgical procedure, resection range, number of lymph node (LN) dissected, tumor location, tumor size, pathological subtype component (including acinar, lepidic, solid, papillary, and micropapillary), presence of lymphovascular invasion (LVI), presence of spread through air space (STAS), and pTNM stage. Tumor size was defined as the maximum diameter of the tumor. Surgical specimens were handled according to standard clinical practice, and the pathology data were retrieved from the pathology report. Histopathological analysis of each specimen was performed by two experienced lung pathologists, following the 2011 classification of the IASLC/ATS/ERS. The histological patterns were identified in 5% increments. The predominant histological subtype was determined based on the pattern with the highest percentage. MPNP-LUAD was defined as lung adenocarcinoma with micropapillary components present but not predominant. Based on good communication with patients, patients chose whether to receive chemotherapy after surgery.

### Patients follow-up

All patients were followed at our outpatient department every three months for the first two years after surgery, and subsequently at six-month intervals. A thoracic and abdominal computerized tomography (CT) scan was routinely performed during each scheduled outpatient department visit for the purpose of follow-up. In the presence of neurological symptoms, a cranial CT scan or magnetic resonance imaging (MRI) was conducted. A positron emission tomography (PET)-CT scan was recommended if possible. Overall survival (OS) was defined as the interval from the date of surgery to either the date of death or the last follow-up. Disease-specific survival (DSS) was defined as the interval from the date of surgery to the date of death specifically caused by lung cancer or the last follow-up. Recurrence-free survival (RFS) was defined as the interval from the date of surgery to the date of the first recurrence or death, or until the last follow-up. The primary endpoint was the 5-year rates of RFS, and the secondary endpoints were the 5-year rates of OS and DSS.

### Statistical analysis

Categorical variables were compared using either the Pearson chi-squared test or Fisher's exact test. Normally distributed continuous variables are typically presented as the mean ± standard deviation (SD), and comparisons were made using Student's t-test. For continuous variables that were not normally distributed, the data are presented as the median (interquartile range [IQR]) and were compared using the Mann–Whitney U test between the groups. To improve the accuracy of comparison between groups, a 1:1 propensity score matching (PSM) analysis was conducted to ensure an equitable distribution of confounding variables between the two groups. Propensity scores were calculated using a multivariate logistic regression model, and a nearest-neighbor matching algorithm was applied without replacement. The variables used to determine PSM were age, sex, smoking history, surgical procedure, resection range, number of lymph nodes dissected, tumor location, tumor size, pathological subtype component, LVI, and STAS.

Kaplan–Meier analyses were performed to compare patients’ survival outcomes, and the log-rank test was used to determine any differences between groups. Moreover, subgroup analyses were performed for the survival outcomes based on the percentage of micropapillary components with a cut-off value of 5%. Finally, univariate and multivariate Cox proportional hazards regression analyses were applied to obtain the hazard ratios (HR) and 95% confidence intervals (CI) of risk factors associated with survival. Multivariate analyses were conducted for risk factors with a *P*-value less than 0.15 that were identified in the univariate analyses. The significance level for the test between the two groups was set at α = 0.05 (two-tailed), and a two-sided P < 0.05 was considered statistically significant. All statistical analyses were performed using SPSS version 25.0 (IBM, Armonk, NY, USA) and R version 4.3.1 (R Development Core Team, Vienna, Austria).

## Results

### Clinicopathological characteristics of patients

**Fig. 1:**
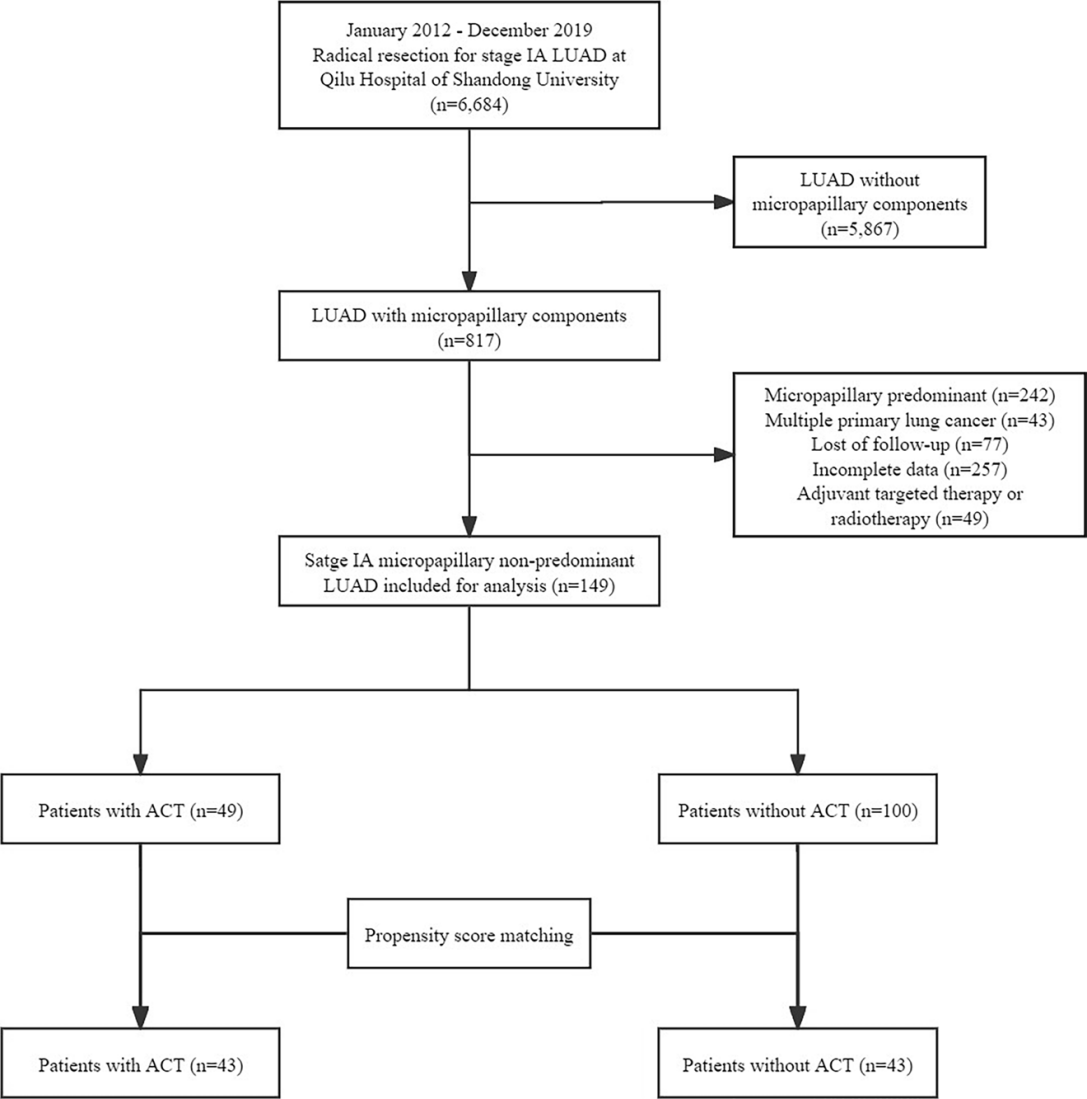
Flow diagram of patient selection through the study. LUAD, lung adenocarcinoma; ACT, adjuvant chemotherapy

During the study period, six thousand six hundred and eighty-four patients underwent surgery for stage IA LUAD at Qilu Hospital of Shandong University, and these patients were screened according to inclusion and exclusion criteria. Finally, a total of 149 patients with comprehensive clinicopathological information and follow-up data were enrolled for further analysis. The detailed flowchart for the population screening process of the cohort is presented in Fig. 1. The included patients were divided into two groups based on their receipt of ACT, among which 49 patients received ACT and 100 patients did not. The clinicopathological characteristics of the patients before and after PSM are presented in Table 1. Before matching, the patients in the two groups were comparable in age, sex, smoking history, surgical procedure, resection range, number of LN dissected, tumor location, tumor size, papillary component, acinar component, lepidic component, LVI, STAS, and pTNM stage (*P* > 0.05). However, there was a significant difference in micropapillary and solid component (*P* < 0.001) between the two groups. Patients who received postoperative ACT tended to have higher micropapillary and solid components. PSM analysis was conducted to ensure a fair distribution of confounding factors between the two groups. And PSM successfully mitigated the heterogeneity observed in the variables between the two groups, as illustrated in Fig. S1. After PSM, 43 pairs of patients were included for analysis, and there were no significant differences in any variables (all *P* > 0.05).

**Table 1:** Clinicopathological characteristics of enrolled patients with stage IA micropapillary non-predominant lung adenocarcinoma before and after PSM

Characteristics	Before PSM			After PSM		
	**Observation (*n* = 100)**	**ACT** **(*n* = 49)**	***P* value**	**Observation (*n* = 43)**	**ACT** **(*n* = 43)**	***P* value**
Age (years), median (IQR)	61.5 (52.3–67.0)	61.0 (52.5–65.0)	0.540	63.0 (51.0–67.0)	61.0 (54.0–65.0)	0.681
Sex, n (%)			0.164			0.276
Female	57 (57.0)	22 (44.9)		16 (37.2)	21 (48.8)	
Male	43 (43.0)	27 (55.1)		27 (62.8)	22 (51.2)	
Smoking history, n (%)			0.271			0.506
Non-smoker	74 (74.0)	32 (65.3)		25 (58.1)	28 (65.1)	
Smoker	26 (26.0)	17 (34.7)		18 (41.9)	15 (34.9)	
Surgical procedure, n (%)			0.584			0.394
Open	13 (13.0)	8 (16.3)		9 (20.9)	6 (14.0)	
VATS	87 (87.0)	41 (83.7)		34 (79.1)	37 (86.0)	
Resection range, n (%)			0.195			1.000
Sublobar resection	14 (14.0)	11 (22.4)		9 (20.9)	9 (20.9)	
Lobectomy	86 (86.0)	38 (77.6)		34 (79.1)	34 (79.1)	
Number of LN dissected, median (IQR)	9.0 (6.0–15.0)	9.0 (6.0–13.5)	0.677	8.0 (5.0–12.0)	9.0 (6.0–13.0)	0.700
Tumor location, n (%)			0.411			0.674
RUL	26 (26.0)	13 (26.5)		12 (27.9)	13 (30.2)	
RML	5 (5.0)	3 (6.1)		1 (2.3)	3 (7.0)	
RLL	32 (32.0)	9 (18.4)		10 (23.3)	7 (16.3)	
LUL	22 (22.0)	12 (24.5)		12 (27.9)	9 (20.9)	
LLL	15 (15.0)	12 (24.5)		8 (18.6)	11 (25.6)	
Tumor size (cm), median (IQR)	2.0 (1.5–2.5)	2.0 (1.5–2.6)	0.955	2.0 (1.5–2.2)	2.0 (1.5–2.6)	0.534
Pathological subtype component (%), median (IQR)						
Micropapillary	5.0 (5.0–10.0)	10.0 (5.0–20.0)	< 0.001	10.0 (5.0–15.0)	10.0 (5.0–20.0)	0.079
Papillary	10.0 (0–60.0)	5.0 (0–40.0)	0.268	9.0 (0–30.0)	0 (0–40.0)	0.879
Solid	0 (0–0)	0 (0–10.0)	< 0.001	0 (0–0)	0 (0–10.0)	0.113
Acinar	40.0 (0.8–80.0)	40.0 (0–80.0)	0.901	70.0 (20.0–90.0)	40.0 (0–80.0)	0.228
Lepidic	0 (0–20.0)	0 (0–10.0)	0.070	0 (0–10.0)	0 (0–10.0)	0.751
Lymphovascular invasion, n (%)			0.260			1.000
No	96 (96.0)	44 (89.8)		40 (93.0)	39 (90.7)	
Yes	4 (4.0)	5 (10.2)		3 (7.0)	4 (9.3)	
Spread through air spaces, n (%)			0.519			0.451
No	82 (82.0)	38 (77.6)		31 (72.1)	34 (79.1)	
Yes	18 (18.0)	11 (22.4)		12 (27.9)	9 (20.9)	
pTNM stage, n (%)			0.387			1.000
IA1	8 (8.0)	2 (4.1)		2 (4.7)	1 (2.3)	
IA2	48 (48.0)	29 (59.2)		25 (58.1)	25 (58.1)	
IA3	44 (44.0)	18 (36.7)		16 (37.2)	17 (39.5)	

*PSM* Propensity score matching, *ACT* Adjuvant chemotherapy, *VATS* Video-assisted thoracoscopic surgery, *LN* Lymph node, *RUL* Right upper lobe, *RML* Right middle lobe, *RLL* Right lower lobe, *LUL* Left upper lobe, *LLL* Left lower lobe, *pTNM* Pathological tumor-node-metastasis, I*QR* Interquartile range

### Kaplan–Meier survival analysis

**Fig. 2:**
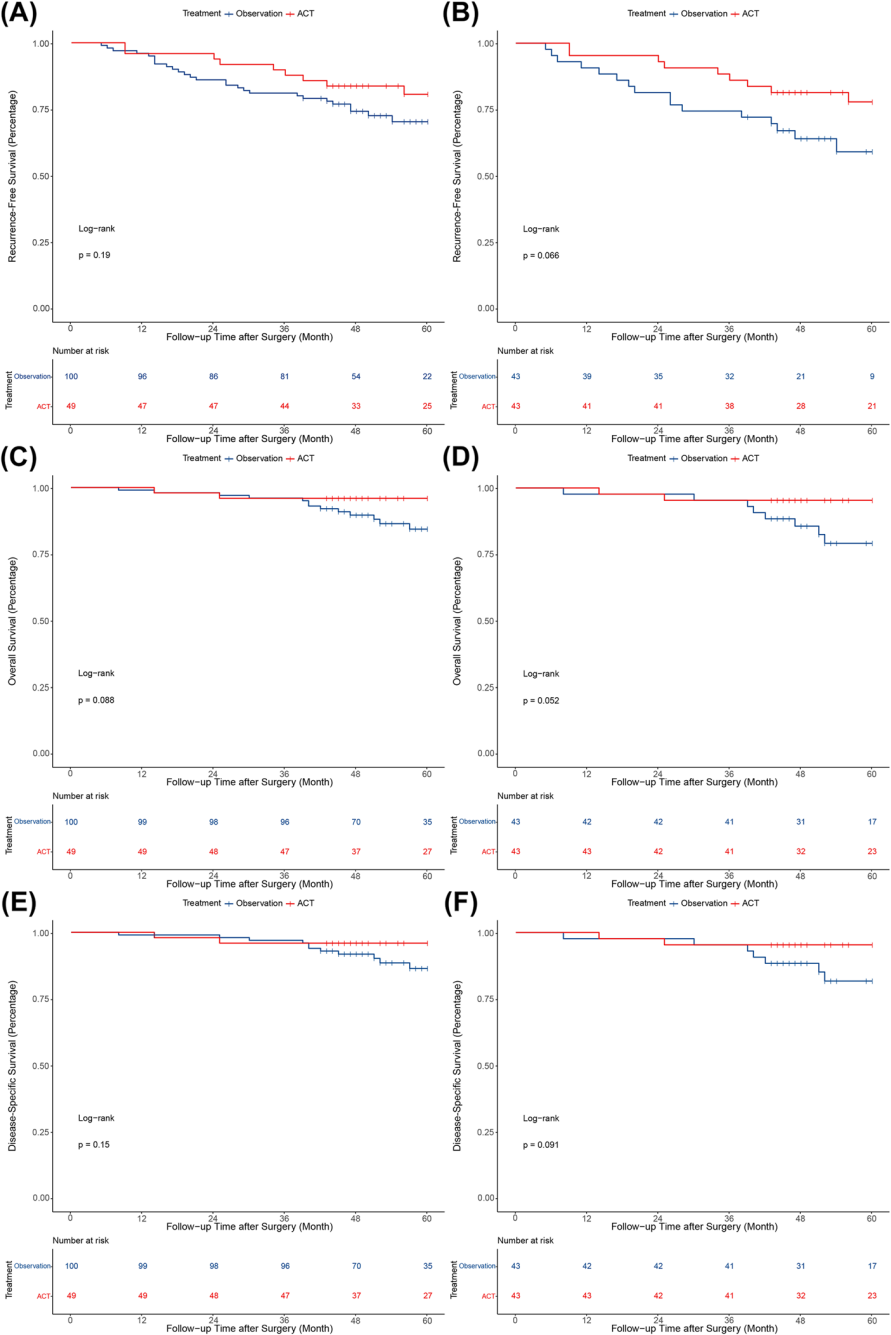
Kaplan–Meier survival curves for all of the included patients. **A** Recurrence-free survival before PSM; **B** Recurrence-free survival after PSM; **C** Overall survival before PSM; **D** Overall survival after PSM; **E** Disease-specific survival before PSM; **F** Disease-specific survival after PSM. PSM, propensity score matching

The median follow-up time for all 149 patients was 54.0 months, ranging from 8.0 to 123.0 months. During the follow-up period, 36 (24.2%) patients experienced recurrence, and 15 (10.1%) patients died. Among the 15 patients who died, 13 died of lung cancer while the other two died of other causes (one died of myocardial infarction, and the other of pulmonary infection). The 5-year rates of RFS, OS, and DSS for all patients were 73.7%, 88.3%, and 89.7%, respectively. Compared to the observation group, patients in the ACT group tended to have higher 5-year RFS rates both before (80.6% vs. 70.2%, P = 0.19) and after PSM (77.9% vs. 59.0%, P = 0.066), but this difference did not reach statistical significance (Fig. 2A-B). As shown in Fig. 2C-D, the 5-year OS rates were 95.9% and 84.3% in the groups with and without ACT before PSM (P = 0.088), and 95.3% and 79.1% after PSM (P = 0.052). And the 5-year rates of DSS were 95.9% and 86.4% in the ACT groups and observation group before PSM (*P* = 0.15), and 95.3% and 81.7% after PSM (*P* = 0.091) (Fig. 2E-F).

### Subgroup analysis based on micropapillary components

**Fig. 3:**
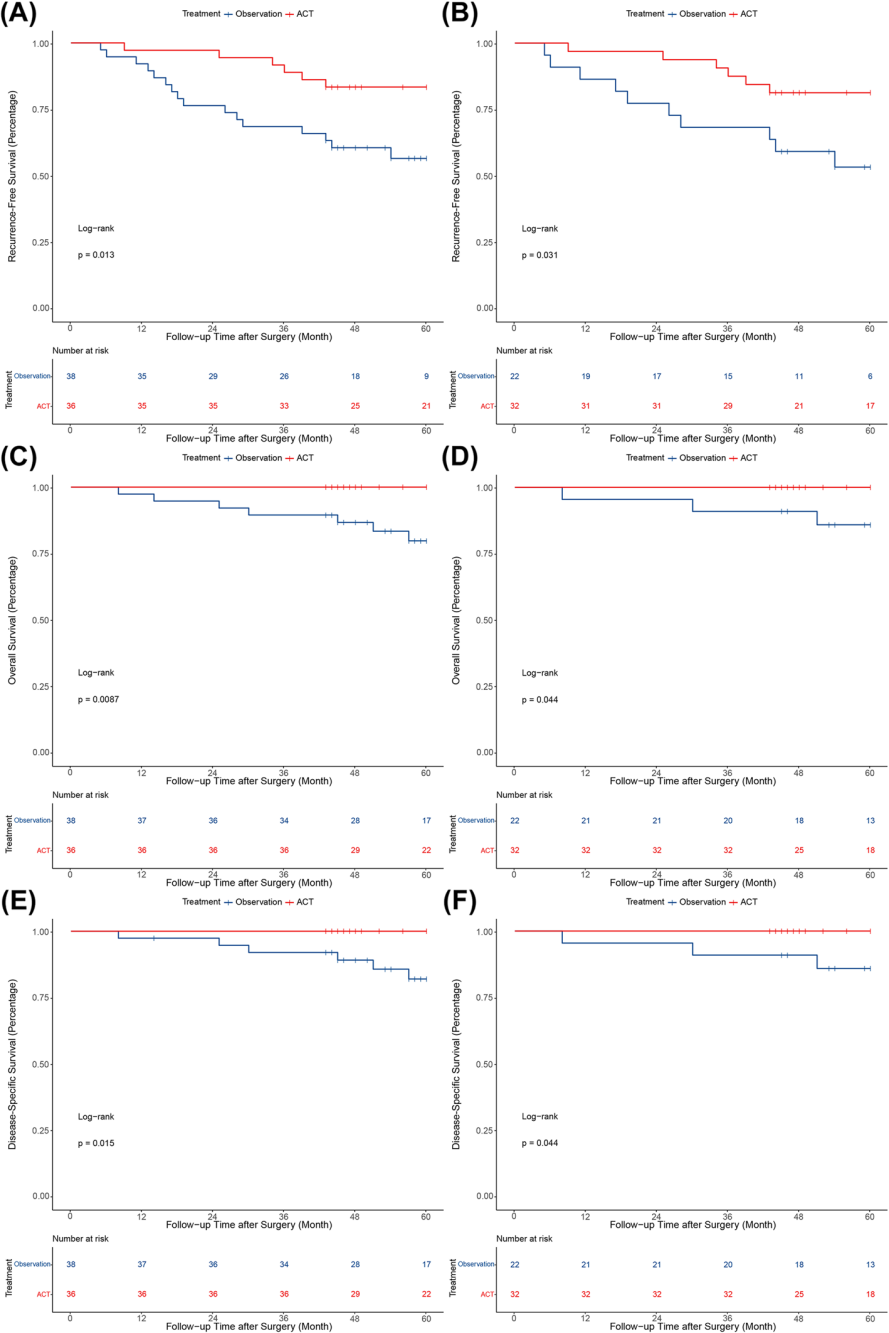
Kaplan–Meier survival curves for patients with a micropapillary component > 5%. **A** Recurrence-free survival before PSM; **B** Recurrence-free survival after PSM; **C** Overall survival before PSM; **D** Overall survival after PSM; **E** Disease-specific survival before PSM; **F** Disease-specific survival after PSM. PSM, propensity score matching

**Fig. 4:**
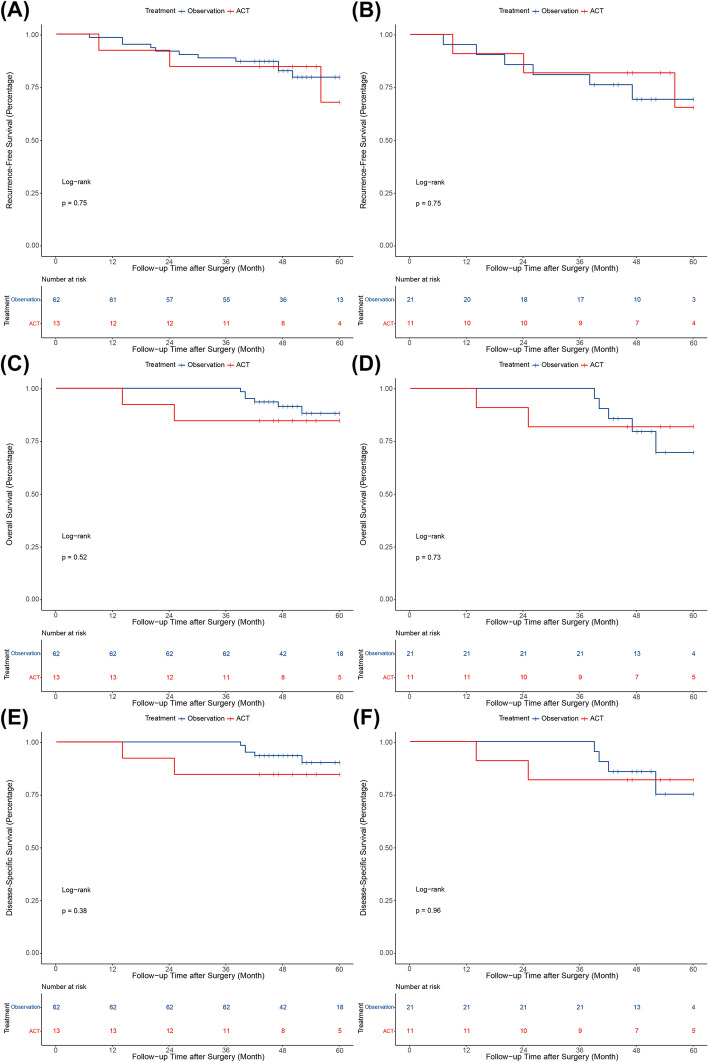
Kaplan–Meier survival curves for patients with a micropapillary component ≤ 5%. **A** Recurrence-free survival before PSM; **B** Recurrence-free survival after PSM; **C** Overall survival before PSM; **D** Overall survival after PSM; **E** Disease-specific survival before PSM; **F** Disease-specific survival after PSM. PSM, propensity score matching

To further explore which specific groups might benefit from postoperative ACT, we performed subgroup analysis. It has been reported that micropapillary components less than or equal to 5% is often defined as sporadic in clinical practice, and may not have an significant prognostic impact. Therefore, a subgroup analysis was performed for the survival outcomes based on the percentage of micropapillary components with a cut-off value of 5%. For patients with a micropapillary component > 5%, the 5-year rates of RFS (83.3% vs. 56.4%, *P* = 0.013), OS (100% vs. 79.7%, *P* = 0.0087), and DSS (100% vs. 81.9%, *P* = 0.015) were significantly higher in the ACT group than in the observation group before PSM (Fig. 3A, C and E). Moreover, the differences in the 5-year RFS (81.3% vs. 53.2%, *P* = 0.031), OS (100% vs. 85.9%, *P* = 0.044), and DSS rates (100% vs. 85.9%, *P* = 0.044) remained significant after PSM (Fig. 3B, D and E). However, for patients with a micropapillary component ≤ 5%, there were no significant differences in the 5-year rates of RFS, OS, and DSS between the two groups, both before and after PSM (all* P* > 0.05) (Fig. 4).

### Cox proportional hazard regression analysis

To further investigate the impact of ACT on patient survival outcomes, we performed Cox proportional hazards regression analysis of RFS, OS, and DSS in patients with a micropapillary component greater than 5%, and the results are presented in Tables 2, 3, and 4 respectively. The prognostic factors identified in the univariate analysis of RFS, including sex (male vs. female; HR = 0.340; 95% CI: 0.133–0.871; P = 0.025), resection range (lobectomy vs. sublobar resection; HR = 0.346; 95% CI: 0.141–0.850; P = 0.021), tumor size (HR = 2.214; 95% CI: 1.073–4.570; *P* = 0.032), and ACT (yes vs. no; HR = 0.325; 95% CI: 0.127–0.832; *P* = 0.019), were incorporated into the multivariate analysis. Afterwards, the resection range (lobectomy vs. sublobar resection; HR = 0.071; 95% CI: 0.020–0.251; P < 0.001), tumor size (HR = 2.929; 95% CI: 1.171–7.330; P = 0.022), and ACT (yes vs. no; HR = 0.122; 95% CI: 0.037–0.403; *P* = 0.001) were identified as independent prognostic factors for RFS through the multivariate analysis. However, the Cox proportional hazard regression analyses failed to identify any significant independent prognostic factors for OS and DSS.

**Table 2:** Cox proportional hazard regression analysis of recurrence-free survival in patients diagnosed with stage IA micropapillary non-predominant lung adenocarcinoma with a micropapillary component > 5%

Variables	Univariate Analysis			Multivariate Analysis		
	**HR**	**95% CI**	***P* value**	**HR**	**95% CI**	***P* value**
Age	0.992	0.950–1.036	0.727			
Sex						
Female	Ref			Ref		
Male	0.340	0.133–0.871	0.025	0.441	0.146–1.332	0.146
Smoking history						
Non-smoker	Ref					
Smoker	0.876	0.323–2.376	0.795			
Surgical procedure						
Open	Ref					
VATS	0.616	0.227–1.671	0.341			
Resection range						
Sublobar resection	Ref			Ref		
Lobectomy	0.346	0.141–0.850	0.021	0.071	0.020–0.251	< 0.001
Surgical side						
Left	Ref					
Right	1.067	0.456–2.498	0.881			
Number of LN dissected	1.007	0.951–1.067	0.804			
Tumor size	2.214	1.073–4.570	0.032	2.929	1.171–7.330	0.022
Micropapillary component	1.034	0.988–1.083	0.152			
Papillary component	1.002	0.989–1.016	0.760			
Solid component	0.987	0.957–1.019	0.432			
Acinar component	0.995	0.982–1.007	0.397			
Lepidic component	1.005	0.992–1.019	0.442			
Lymphovascular invasion						
No	Ref					
Yes	0.581	0.078–4.329	0.597			
Spread through air spaces						
No	Ref					
Yes	0.837	0.247–2.837	0.775			
pTNM stage						
IA1	Ref					
IA2	6990.189	0–6.386E + 80	0.922			
IA3	12,719.830	0–1.162E + 81	0.917			
Adjuvant chemotherapy						
No	Ref			Ref		
Yes	0.325	0.127–0.832	0.019	0.122	0.037–0.403	0.001

*VATS* Video-assisted thoracoscopic surgery, *LN* Lymph node, *pTNM* Pathological tumor-node-metastasis, *HR* Hazard Ratio, *CI* Confidence interval, *Ref* Reference

**Table 3:** Cox proportional hazard regression analysis of overall survival in patients diagnosed with stage IA micropapillary non-predominant lung adenocarcinoma with a micropapillary component > 5%

Variables	Univariate Analysis			Multivariate Analysis		
	**HR**	**95% CI**	***P* value**	**HR**	**95% CI**	***P* value**
Age	1.141	1.012–1.286	0.032	1.113	0.980–1.265	0.100
Sex						
Female	Ref					
Male	0.801	0.179–3.583	0.772			
Smoking history						
Non-smoker	Ref					
Smoker	0.528	0.064–4.389	0.555			
Surgical procedure						
Open	Ref					
VATS	0.513	0.099–2.647	0.425			
Resection range						
Sublobar resection	Ref					
Lobectomy	1.053	0.127–8.755	0.962			
Surgical side						
Left	Ref					
Right	2.142	0.413–11.108	0.364			
Number of LN dissected	1.035	0.944–1.134	0.467			
Tumor size	5.601	1.201–26.114	0.028	4.591	0.931–22.635	0.061
Micropapillary component	0.968	0.873–1.072	0.533			
Papillary component	1.000	0.975–1.026	0.986			
Solid component	0.917	0.703–1.197	0.525			
Acinar component	0.973	0.943–1.004	0.087	0.982	0.945–1.020	0.350
Lepidic component	1.026	1.007–1.046	0.007	1.015	0.992–1.038	0.208
Lymphovascular invasion						
No	Ref					
Yes	0.045	0–71934.377	0.670			
Spread through air spaces						
No	Ref					
Yes	0.039	0–1043.832	0.533			
pTNM stage						
IA1	Ref					
IA2	5281.969	0–2.541E + 149	0.960			
IA3	17,160.754	0–8.243E + 149	0.955			
Adjuvant chemotherapy						
No	Ref					
Yes	0.016	0.000–6.789	0.179			

*VATS* Video-assisted thoracoscopic surgery, *LN* Lymph node, *pTNM* Pathological tumor-node-metastasis, *HR* Hazard Ratio, *CI* Confidence interval, *Ref*. Reference

**Table 4:** Cox proportional hazard regression analysis of disease-specific survival in patients diagnosed with stage IA micropapillary non-predominant lung adenocarcinoma with a micropapillary component > 5%

Variables	Univariate Analysis			Multivariate Analysis		
	**HR**	**95% CI**	***P* value**	**HR**	**95% CI**	***P* value**
Age	1.097	0.977–1.231	0.119	1.066	0.946–1.203	0.294
Sex						
Female	Ref					
Male	0.533	0.097–2.910	0.467			
Smoking history						
Non-smoker	Ref					
Smoker	0.630	0.074–5.393	0.673			
Surgical procedure						
Open	Ref					
VATS	0.413	0.075–2.259	0.308			
Resection range						
Sublobar resection	Ref					
Lobectomy	0.876	0.102–7.506	0.904			
Surgical side						
Left	Ref					
Right	1.752	0.319–9.634	0.519			
Number of LN dissected	1.033	0.935–1.141	0.526			
Tumor size	4.276	0.888–20.591	0.070	3.491	0.636–19.151	0.150
Micropapillary component	0.979	0.884–1.085	0.689			
Papillary component	0.981	0.942–1.022	0.366			
Solid component	0.917	0.687–1.223	0.555			
Acinar component	0.976	0.946–1.007	0.131	0.997	0.954–1.042	0.879
Lepidic component	1.032	1.010–1.055	0.004	1.028	0.996–1.061	0.086
Lymphovascular invasion						
No	Ref					
Yes	0.045	0–3.003E + 5	0.699			
Spread through air spaces						
No	Ref					
Yes	0.039	0–3098.496	0.574			
pTNM stage						
IA1	Ref					
IA2	5858.593	0–2.722E + 155	0.961			
IA3	15,305.930	0–7.102E + 155	0.957			
Adjuvant chemotherapy						
No	Ref					
Yes	0.016	0.000–11.028	0.214			

*VATS* Video-assisted thoracoscopic surgery, *LN* Lymph node, *pTNM* Pathological tumor-node-metastasis, *HR* Hazard Ratio, *CI* Confidence interval, *Ref* Reference

## Discussion

The 2011 classification by the IASLC/ATS/ERS introduced a categorization system for invasive LUAD based on distinct histological subtypes, which significantly contributes to predicting the prognosis for patients with LUAD [5, 18]. It has been reported that MPP subtype was associated with an increased risk of lymph node metastasis, pleural invasion, and a more unfavorable prognosis [19]. Wang et al. found that patients with MPP-LUAD in stage IA might benefit from ACT [16]. In clinical practice, the MPNP pattern is more common than MPP, especially in the early stages. A meta-analysis indicated that the presence of a micropapillary component in stage IA LUAD, regardless of the level, was associated with an increased risk of recurrence [10]. The prognostic significance of ACT for patients with stage IA MPNP-LUAD remains unknown until today. In the present study, we found that postoperative ACT might provide survival benefits for patients with stage IA MPNP-LUAD who have a micropapillary component greater than 5%. To the best of our knowledge, this study is the first to assess the impact of ACT on patients diagnosed with stage IA MPNP-LUAD.

The presence of micropapillary components in stage IA LUAD patients is a prognostic risk factor, and many previous studies have focused on the prognostic impact of the percentage of micropapillary components [20, 21]. It has been reported that a micropapillary component of 5% or less might not be sufficient to affect the prognosis of patients. Su et al. found that in LUAD patients with a tumor size ≤ 2 cm, there was no difference in prognosis between patients with a micropapillary component less than 5% and those with a micropapillary component equal to 5%. With the increase of the proportion of micropapillary components, its influence on prognosis became more apparent. However, when the proportion of micropapillary components reaches a threshold value (approximately 20%), the impact of its increase on prognosis becomes less significant [22]. Similarly, Tsubokawa et al. found that patients with a micropapillary component > 5% had a significantly worse 5-year disease-free survival (DFS) than those with a micropapillary component ≤ 5%, but there was no significant difference in 5-year DFS rates between patients with a micropapillary component ≥ 30% and those with a micropapillary component between 5 and 25% [23]. Therefore, we supposed that patients with micropapillary components greater than 5% would have a poorer prognosis and might benefit from postoperative adjuvant therapy.

Previous studies have shown limited efficacy of ACT in patients undergoing radical resection of stage IA LUAD, in part because of the inclusion of patients with a better prognosis who may not require the treatment. Therefore, it is necessary to screen out the high-risk population with high-risk pathological factors before exploring the role of ACT. Sasada et al. found that, after excluding preinvasive lesions and lepidic predominant LUAD in stage IA, the 5-year OS of the ACT group was better than that of the control group [24]. Other studies have suggested that patients with a micropapillary predominant pattern or poorly differentiated tumors in stage IA may benefit from ACT [16, 25]. In this study, we found that postoperative ACT might provide a better survival for patients with stage IA MPNP-LUAD who have a micropapillary component greater than 5%, while patients with a micropapillary component ≤ 5% might not benefit from ACT. In addition, the Cox proportional hazard regression analysis also identified ACT as independent favorable prognostic factors for RFS in patients with stage IA MPNP-LUAD who have a micropapillary component > 5%. However, we found that ACT was not associated with a better OS or DSS in the Cox regression analysis, which could be attributed to the small sample size and the better survival outcomes of the patients.

Unexpectedly, the Cox proportional hazard regression analysis identified lobectomy as an independent favorable prognostic factor for RFS in patients with stage IA MPNP-LUAD who have a micropapillary component greater than 5%. Similarly, a study showed that segmentectomy had a significantly worse prognosis than lobectomy when the micropapillary components exceeded 5% [22]. Therefore, our findings suggested that lobectomy should be potentially recommended for this group of patients.

This study has several limitations that should be taken into consideration. Firstly, the retrospective nature of this study being conducted at a single center diminishes its persuasiveness compared to a multicenter prospective randomized controlled trial. Second, the small sample size of this study weakened our conclusions to some extent. Third, despite the use of PSM to mitigate the influence of confounders among the two groups, it is necessary to note that potential selection bias was not completely eliminated. Furthermore, the regimens, dosages, and toxicity of patients who received ACT have not been evaluated. Finally, we did not analyze how patients were treated after recurrence, which might have an impact on their OS and DSS. Multi-center, prospective randomized controlled trials with a large sample size are required to validate our findings.

## Conclusion

Patients with stage IA MPNP-LUAD who have a micropapillary component greater than 5% might experience improved survival outcomes when receiving ACT after surgery. However, patients with a micropapillary component equal to or less than 5% did not appear to derive the same benefit from postoperative ACT. Postoperative ACT was potentially recommended for stage IA MPNP-LUAD patients with a micropapillary component greater than 5%.

### Supplementary Information

**Additional file 1. Fig. S1.** Evaluation of outcomes of propensity score matching (PSM) analysis.

## Data Availability

No datasets were generated or analysed during the current study.

## Abbreviations

NSCLC: Non-small cell lung cancer
LUAD: Lung adenocarcinoma
IASLC: International Association for the Study of Lung Cancer
ATS: American Thoracic Society
ERS: European Respiratory Society
ACT: Adjuvant chemotherapy
MPP: Micropapillary predominant
MPNP: Micropapillary non-predominant
pTNM: Pathological tumor-node-metastasis
IRB: Institutional Review Board
LN: Lymph node
LVI: Lymphovascular invasion
STAS: Spread through air space
CT: Computerized tomography
MRI: Magnetic resonance imaging
PET: Positron emission tomography
OS: Overall survival
DSS: Disease-specific survival
RFS: Recurrence-free survival
SD: Standard deviation
IQR: Interquartile range
PSM: Propensity score matching
HR: Hazard ratios
CI: Confidence intervals
