# Supplementary figures and images for: Prognostic significance and survival benefits of postoperative adjuvant chemotherapy in patients with stage IA lung adenocarcinoma with non-predominant micropapillary components
Source: World J Surg Oncol. 2024 Jan 25;22:32. doi: 10.1186/s12957-024-03303-x (PMC10809442; doi:10.1186/s12957-024-03303-x)

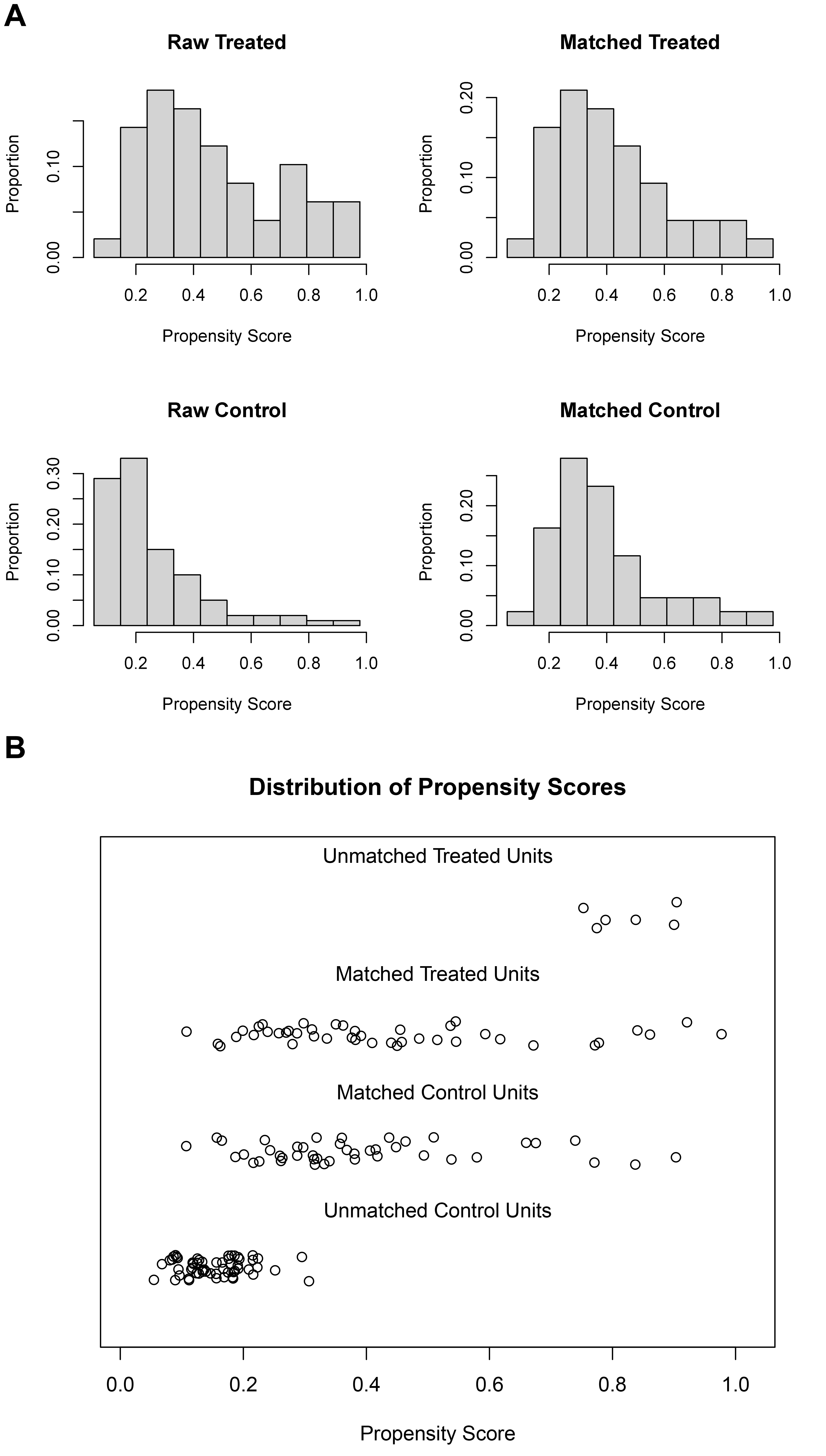

Supplement: Supplementary file 1 — Additional file 1. Fig. S1. Evaluation of outcomes of propensity score matching (PSM) analysis. [file 12957_2024_3303_MOESM1_ESM.tif]
